# Towards a Treatment for Leukodystrophy Using Cell-Based Interception and Precision Medicine

**DOI:** 10.3390/biom14070857

**Published:** 2024-07-17

**Authors:** Benoit Coulombe, Alexandra Chapleau, Julia Macintosh, Thomas M. Durcan, Christian Poitras, Yena A. Moursli, Denis Faubert, Maxime Pinard, Geneviève Bernard

**Affiliations:** 1 Translational Proteomics Laboratory, Institut de Recherches Cliniques de Montréal, Montréal, QC H2W 1R7, Canada; christian.poitras@ircm.qc.ca (C.P.); yena.moursli@ircm.qc.ca (Y.A.M.); maxime.pinard@ircm.qc.ca (M.P.); 2 Department of Biochemistry and Molecular Medicine, Université de Montréal, Montréal, QC H3T 1A8, Canada; 3 Department of Neurology and Neurosurgery, Pediatrics and Human Genetics, McGill University, Montréal, QC H9X 3V9, Canada; alexandra.chapleau@mail.mcgill.ca (A.C.); julia.macintosh@mail.mcgill.ca (J.M.); genevieve.bernard@mcgill.ca (G.B.); 4 Child Health and Human Development Program, Research Institute of the McGill University Health Centre, Montréal, QC H4A 3J1, Canada; 5 The Neuro’s Early Drug Discovery Unit (EDDU), McGill University, Montréal, QC H9X 3V9, Canada; thomas.durcan@mcgill.ca; 6 Mass Spectrometry and Proteomics Platform, Institut de Recherches Cliniques de Montréal, Montréal, QC H2W 1R7, Canada; denis.faubert@ircm.qc.ca; 7 Department Specialized Medicine, Division of Medical Genetics, McGill University Health Centre, Montréal, QC H4A 3J1, Canada

**Keywords:** cell-based interception and precision medicine, leukodystrophy, POLR3-related leukodystrophy (POLR3-HLD), proteomics, single-cell technologies, SCoPE2-MS, induced pluripotent stem cells

## Abstract

Cell-based interception and precision medicine is a novel approach aimed at improving healthcare through the early detection and treatment of diseased cells. Here, we describe our recent progress towards developing cell-based interception and precision medicine to detect, understand, and advance the development of novel therapeutic approaches through a single-cell omics and drug screening platform, as part of a multi-laboratory collaborative effort, for a group of neurodegenerative disorders named leukodystrophies. Our strategy aims at the identification of diseased cells as early as possible to intercept progression of the disease prior to severe clinical impairment and irreversible tissue damage.

## 1. Introduction

Under the leadership of Niklaus Rajewski from the Max Delbruck Center in Berlin, Germany and Geneviève Almouzni from the Curie Institute in Paris, France, the Lifetime initiative was launched to support the development of cell-based interceptive medicine [1]. The goal of the LifeTime initiative is to generate tools, reagents, and technologies to measure, analyze, and predict the mechanisms involved in disease onset and progression. LifeTime is expected to expand our basic understanding of genome function within cells and tissues, significantly influencing basic biomedical sciences across diverse fields. In addition, this initiative is likely to redefine the role of artificial intelligence (AI) in precision medicine by acting on cell and molecular biology [2]. For example, some LifeTime scientists are developing novel AI-driven cellular imaging software, while others harness AI/AlphaFold software (version 2.3.2) for molecular modeling. LifeTime is expected to design 21st century biology and next-generation data sciences that will directly and profoundly impact medical practice, improving human health by intervening early in disease progression. Clearly, the new developments in the health sector will provide benefits in terms of business volume, jobs, and personal income. In addition, several pharmaceutical companies will be strengthened by the emergence of totally novel targeted medicines. Indeed, a large number of pharmaceutical companies, as well as many biomedical research centers, have already initiated partnerships with LifeTime in Europe.

The 37TrillionCells initiative has been created by Canadian researchers to accelerate the development of cell-based interception and precision medicine by taking advantage of and further developing novel proteomics technologies at single-cell resolution [3]. The 37TrillionCells initiative is currently under the leadership of Benoit Coulombe from the Institut de Recherches Cliniques de Montréal and Université de Montréal. Details regarding this Montréal-based proteomics initiative are provided in this manuscript and represent the core of the 37TrillionCells initiative. The 37Trillion Cells initiative seeks to provide a proteomics-specific approach aimed at complementing LifeTime’s specific objectives.

**Figure 1:**
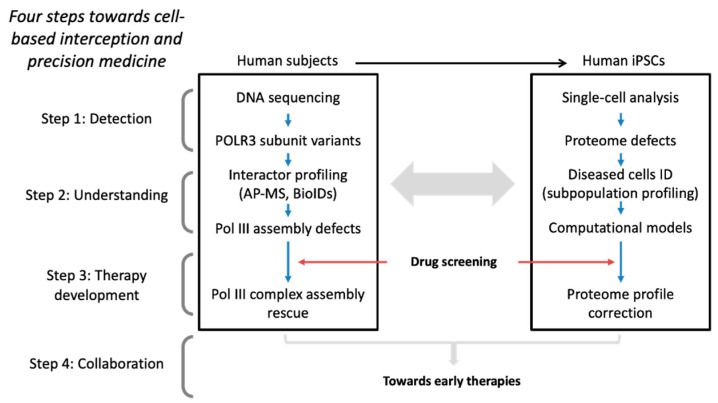
Scheme for cell-based interception and precision medicine applied to leukodystrophies.

Cell-based interception and precision medicine is an emerging medical paradigm where diseased cells are identified and intercepted as early as possible, before symptoms present and irreversible tissue damages occurs, enabling the development of therapies for the early treatment of diseases [1,2,3]. One of the most challenging aspects of this approach is developing ultra-sensitive and reproducible methods for diseased cell detection. Detection through DNA sequencing of variants in known disease-causing genes is one way to achieve early diagnosis. As mutations are present in all cells in every developmental stage, even before birth, identifying cells that show increased vulnerability to potential pathogenic variants and are specifically involved in disease progression requires additional analyses. For example, characterization of disease-causing variants using affinity purification coupled with mass spectrometry (AP-MS) has been exploited by looking at changes in interaction partner profiles [4,5,6]. However, interactor profiling using AP-MS falls short of specifically identifying diseased cells. Single-cell transcriptomics and proteomics have revolutionized the ability to profile gene/protein expression networks allowing early identification of dysfunction and disease manifestations. Once affected cells are detected, disease mechanisms can be elucidated using experimental cell models and predictive computational cell trajectory modeling [7]. This approach can identify proteins (including posttranslational modifications, PTMs), protein complexes, and intra- or extra-cellular networks that participate in the disease process and provides the necessary knowledge for the development of disease-modifying therapies. This procedure can be briefly summarized into four steps: detection, understanding, therapy development, and lastly, the implementation of collaborative work to form a multidisciplinary team of experts (Figure 1). As a prototype of step 4, we have initiated the development of a procedure applied to hypomyelinating leukodystrophies.

Leukodystrophies are a group of hereditary white matter disorders that predominantly affect children, causing a gradual decline in abilities and often resulting in early mortality within months to years after onset [8]. These conditions selectively affect the brain’s white matter, which serves as the protective covering for nerve cells within the brain and spinal cord. Ultimately, this results in abnormalities in myelin formation (classified as hypomyelinating) or disruptions in myelin maintenance (non-hypomyelinating) which compromises the proper functioning of the central nervous system (CNS) leading to neurodegeneration and ensuing sequalae [9,10,11]. To date, there are over 50 different well-defined leukodystrophies with diverse clinical and genetic characteristics and varied Mendelian inheritance patterns. A myriad of genes involved in disparate cellular processes are known to cause leukodystrophies, including those involved in brain development and functioning, metabolism, and housekeeping functions to name a few. Most leukodystrophies remain without a cure or disease-modifying therapy, and interventions are primarily focused on symptomatic treatment [12,13]. Here, we examine the benefits of cell-based interception and precision medicine procedures to hypomyelinating leukodystrophies, with a focus on RNA polymerase III-related leukodystrophy. 

## 2. Cell-Based Interception and Precision Medicine Applied to Leukodystrophies

### 2.1. Detect

Sequence analysis of the genome or exome of patients and their parents can reveal the presence of pathogenic variant(s) causing a leukodystrophy. In the last few years, our group has identified and validated eight genes causative for leukodystrophy, including *POLR3A*, *POLR3B*, *POLR1C*, *POLR3D*, *EPRS1*, *VARS1*, and *ABHD16A* [4,14,15,16,17,18,19]. In tandem, we have developed a complimentary rare disease database to collect detailed information on individual patients’ phenotypes, positioning our group in a unique capacity to integrate genetic, clinical, and pathophysiologic research to better advance patient care and eventual treatment of these disorders [20]. 

**Figure 2:**
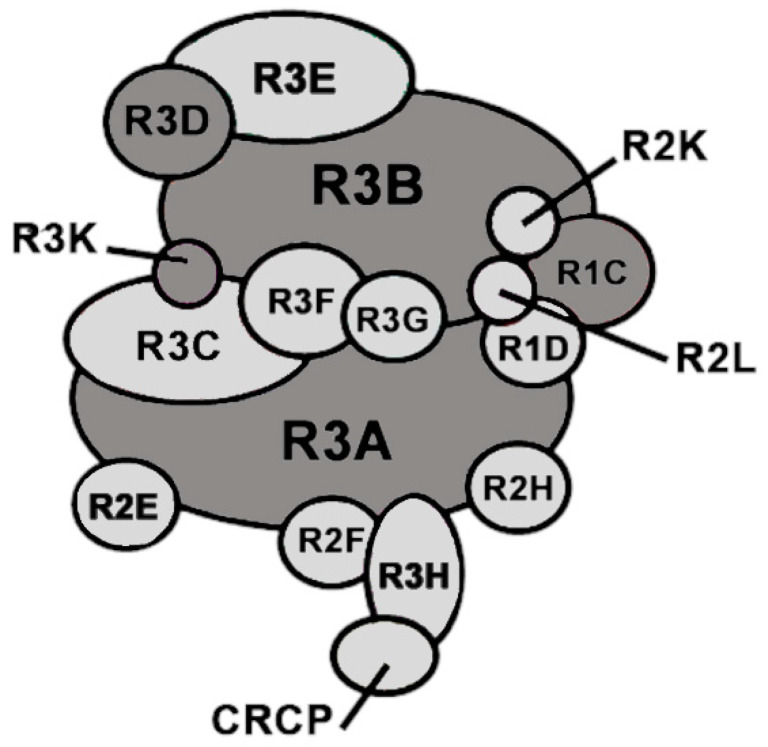
Schematic representation of the 17-subunit Pol III complex. Leukodystrophy-causative mutations were found in the subunits marked in dark grey.

Specifically, our group has identified four of the five known causative genes associated with POLR3-related leukodystrophy (POLR3-HLD), one of the most common hypomyelinating leukodystrophies [21]. POLR3-HLD, also termed 4H (Hypomyelination, Hypodontia, and Hypogonadotropic Hypogonadism) leukodystrophy, is caused by biallelic pathogenic variants in genes encoding subunits of the ubiquitous RNA polymerase III (Pol III) enzymatic complex (e.g., *POLR3A*, *POLR3B*, *POLR1C*, *POLR3K*, and *POLR3D*) [4,14,15,16,22] (Figure 2). The majority of variants are found in the genes encoding the two largest subunits of Pol III, *POLR3A* and *POLR3B*, which form the catalytic core of the complex [23]. Leukodystrophy-associated variants in genes encoding Pol III subunits have been shown to either reduce the amount of a given subunit, to cause abnormal interactions of the affected subunit with other Pol III subunits, impacting proper complex assembly, or to interfere with the enzymatic activity involved in the transcription of small non-coding RNAs [4,24,25]. Indeed, crystallization of the human Pol III complex in the past few years confirmed that the majority of pathogenic variants in POLR3-HLD map to subunit interfaces or DNA-binding domains, further highlighting how some of these variants may impact Pol III complex assembly or its transcriptional activity [26,27].

Pathogenic variants of genes encoding aminoacyl-tRNA synthetases (ARS) have been linked to a number of neurodegenerative diseases, including hypomyelinating leukodystrophies (e.g., *EPRS1* [17], *VARS1* [18], *DARS1* [28] and *RARS1* [29]). Further, biallelic pathogenic variants in *POLR1A* also cause leukodystrophy [30,31], further supporting the link between translation (i.e., protein production) and CNS development. Although genotype–phenotype correlations exist for some pathogenic variants [23,32,33,34], this is not the case for most patients, thereby necessitating further analyses beyond the genetic basis to successfully stratify patient cohorts. In addition, the identification of disease-causing variants does not provide much insight into potential disease mechanisms or identify the cells and pathways most affected. To this end, analysis of gene/protein expression profiles at single-cell (SC) resolution appears necessary to identify the cells most vulnerable to disease and may prove to be a useful tool for patient stratification and treatment. SC transcriptomics and proteomics allow the ability to profile gene expression and identify the top dysfunctional cell candidates. 

### 2.2. Understand

Despite having shown that Pol III complex assembly defects are one of the most common outcomes of pathogenic variants in POLR3-HLD, questions remain as to how they lead to hypomyelination. Recent in vivo and in vitro models of POLR3-HLD have found impaired oligodendrocyte (OL) development, pointing to a cellular mechanism for the hypomyelination seen in disease [35,36,37]. Recently, a severe conditional/inducible mouse model with an exon 10 deletion in *Polr3b* (*Polr3b∆10*) was shown to recapitulate characteristic neurological and non-neurological features of disease, including hypomyelination [35]. Lineage tracing analysis in these mice revealed insufficient mature OLs and a reduced proliferative capacity of OL precursor cells (OPCs), highlighting the cellular defects underlying this hypomyelination [35]. A less severe mouse model with a conditional knock-in of two adjacent variants in *Polr3a* also showed a reduced number of mature MOG+ OLs, emphasizing an impairment of oligodendrogenesis in POLR3-HLD [36]. Further, an in vitro approach based on downregulating the endogenous expression of leukodystrophy-associated Pol III subunits via siRNA found an impairment of OL maturation, most evident at the level of reduced morphological complexity, in addition to impaired myelination capacity [37]. Nonetheless, additional work is required to link impaired Pol III function to stunted OL development. In fact, two hypotheses have been proposed to explain the vulnerability of OLs to disease [11]. First, some authors have hypothesized that defects in Pol III function likely affect a key as-yet-unidentified component of myelin biogenesis or the maintenance apparatus. For instance, Choquet et al. found that the M852V mutation in *POLR3A* specifically downregulates the expression of the Pol III transcript *BC200*, a gene encoding a brain cytoplasmic regulatory RNA [5]. So far, a function for *BC200* in regulating myelination has not been conclusively established. The second hypothesis pertains to the protein synthesis demand required for OL development and myelination. OLs and their progenitors necessitate that a large amount of protein be produced to proliferate, mature, and expand their processes, and to myelinate the brain [38]. As such, defects in Pol III transcription, by impairing the production of components of the translation machinery (e.g., transfer RNAs (tRNAs) and the 5S ribosomal RNA (rRNA)), may have a deleterious effect on this particular process. This second hypothesis is supported by recent work, which identified a decrease in distinct tRNAs in Pol III siRNA-treated OLs [37]. This reduction of specific tRNA transcripts has likewise been shown in patient-derived fibroblasts [16,22]. Moreover, the fact that other hypomyelinating disorders are associated with pathophysiological mechanisms involving abnormal translation (including the aforementioned ARS; see for example Mendes et al. [17] and references therein) further substantiates this latter hypothesis [17,28]. Finally, it is possible that these hypothesized mechanisms are not mutually exclusive and that both contribute to disease pathogenesis.

To understand the effect of Pol III subunit variants in human cells, we used affinity purification coupled with mass spectrometry (AP-MS) as a highly sensitive proteomic method to assess polymerase assembly. In several cases, we observed Pol III assembly defects and/or impact on its assembly with co-factors [4,16,25]. In those that were further studied, these assembly defects proved to decrease transcription efficiency by the polymerase [4] or were shown to be embryonically lethal in a homozygote mouse model [25]. A limitation of these proteomic studies is that they have not investigated the diseased cells specifically. Further experiments, including single-cell proteomics (SCP), are required to further understand the molecular mechanisms of this disease. 

Using a procedure adapted from the SCoPE2-MS technique developed in the Slavov laboratory at Northeastern University, USA [39,40,41], multiple sample preparation steps can be combined to minimize sample manipulation, resulting in reduced peptide loss. It incorporates Tandem Mass Tag (TMT) as a chemical barcode enabling the simultaneous detection of multiple single cells, facilitating analysis of high quantities of specific proteins Each single cell is labeled with a TMT-10plex (Fisher scientific, Hampton, New Hampshire), but the TMT-labeled pool also includes important controls such as a carrier (200 cells of interest), a reference (5 cells of interest) and a negative control (no cells). In the future, we anticipate that additional TMT tags will be developed, further increasing the number of analyzed cells. We plan to improve our procedure with the TMT18plex/proteome discoverer combo. Sample analysis is performed on a Fusion Orbitrap MS, and raw data are analyzed as in Specht et al., 2021 [41] by using a combination of MaxQuant (version 2.4.13.0) [42] and DO-MS analysis using the R programming language (version 2.0.8) [43]. MaxQuant is used to match peptides to the proper characterized proteins in the UniProtKB database [44], with TMT tags as fixed modifications and additional parameters. Subpopulation evaluation will be assessed by performing a principal component analysis (PCA) [45].

To increase specificity and disease relevance, we plan to perform our SCP profiling using induced pluripotent stem cells (iPSCs) and their downstream derivatives [46]. Utilizing iPSCs provides the benefits of being able to directly study disease-relevant patient tissue as they can be reprogrammed directly from patients and directed to differentiate into cell types of interest. In the case of hypomyelinating leukodystrophies, examining CNS cell types, notably glial cells, would be most specific to our platform. Using a recent adaptation of a widely used protocol to derive OPCs [47], we can generate mixed glial cultures of astrocytes, OPCs, and MBP+ OLs [48], thereby limiting the use of bulk analysis. Thus, fluorescent-activated cell sorting (FACS) is essential to isolate the specific cell types to be profiled by SCP using different combinations of cellular markers identified by our group [47,49]. Proteomic profiling of a high number of single cells will provide a better understanding of the molecular function and the developmental stage and trajectory of diseased cells. Further, these results will provide complementary data to publicly available SC transcriptomics sequencing [50,51,52] as well as unpublished sequencing data of our iPSC-derived glial cells. 

Indeed, single-cell transcriptomics (also known as sc-RNAseq) has been invaluable not only to study the progression of different diseases, but also to study the biology of various tissues and organs including the CNS. SC transcriptomics studies performed in either mouse brain tissues or human iPSCs have confirmed the heterogeneity of OPC, OL, and astrocyte populations [50,51,52,53,54,55,56,57]. These different populations were confirmed at the single-cell resolution level after the analysis of specific cells obtained either at different developmental stages of the CNS [50,52,53,55,56], at localization in brain tissue sections [56,57], or in multiple disease cells [54,56]. The subpopulations observed so far contained multiple clusters with a range between immature OPCs to mature myelinating OLs. Moreover, the expression of different genes such as *PDGFRα*, *Nkx2-2*, *SOX10*, *PLP1*, and *MBP,* to name a few, was associated with specific cell population clusters and could be used to follow these populations during differentiation in various experimental conditions [50,52]. 

Nonetheless, these variations do not indicate protein fate or which cascades are activated during cell maturation. Because proteins are the functional actors of cells, and because RNA levels often do not correlate with protein levels, we think that single-cell proteomics (SCP) is a method of choice to profile cells in a given tissue. Proteomic studies are likely to provide details beyond sc-RNAseq, which can include protein stability, cellular localization, or more importantly, posttranslational modifications (PTMs). For example, the phosphorylation, methylation, acetylation, ubiquitination, and proteolytic cleavage profiles of peptides can be differentially detected in different cells. Researchers have identified more than 400 different types of PTMs that regulate the function of proteins [58]. We expect to establish roles for some of them during brain cell differentiation or disease progression. In addition, the stoichiometry of protein components that are part of given complexes is likely to determine whether whole complexes or individual subunits are regulated during cell differentiation or disease progression. For example, if RNA polymerase III (or any other multisubunit complex) is regulated during the differentiation of a given cell type, we expect to find a difference in the expression of multiple polymerase subunits in our analysis. These previous points are in addition to the changes in protein expression levels occurring during normal or disease cell evolution. In the case of leukodystrophies, we trust that changes in the proteome during disease progression will illuminate causative mechanisms and reveal the link between Pol III defects and aberrant myelin metabolism. Such a discovery would represent a quantum leap in our understanding of the causative mechanisms of leukodystrophies.

To advance the understanding of molecular defects, we intend to employ SCP to map the proteomic profiles of the cells most vulnerable to leukodystrophy-causative variants. Recent progress in the development of proteomics and microfluidic and computational methods has allowed the detection of 100s to 1000s of proteins in single cells [59,60,61]. However, most of them are abundant proteins, and the detection of low-abundance proteins is still a challenge. We have recently developed a procedure for systematic identification of low-abundance proteins in specific parts of the proteome. This method generates a library of low-abundance MS2 peptides with varying levels in disease vs. healthy conditions. Monitoring these peptides allows the understanding of the role of each protein at different stages in diseased cells and the evaluation of their response to drug treatment. This generic method is applicable to any type of disease, making it a generalizable platform to elucidate disease progression mechanisms and accelerate drug and biomarker discovery. Peptides from the list corresponding to known proteins of OLs and OPCs [62,63] are validated for their capacity to discriminate between OLs or OPCs by conducting additional SCP experiments. scRNA-seq experiments will be used to support the proteomic profile difference observed between OLs and OPCs. These validation experiments will serve to monitor proteome changes during diseased cell progression through cell differentiation into OLs, in organoid spatial organization, or after drug treatment. Such a targeted approach using a disease-specific (or condition-specific) peptide library, which we named Deep-SCP, is expected to greatly increase the sensitivity and throughput of our new improved method.

In the future, we aim to test this strategy in myelinating organoids to expand our understanding of disease using 3D models [64]. Overall, iPSC-derived CNS cells represent an interesting alternative to recapitulate brain tissue and could be used to assess the impact of pathogenic variants on disease development and advance potential therapeutics. 

### 2.3. Therapy Development

The unraveling of dysfunctions in Pol III assembly caused by pathogenic variants in genes encoding different subunits prompted us to evaluate the effect of drugs known to modulate the function of molecular chaperones and general proteostasis factors, to improve Pol III assembly and function [65,66]. A number of small molecules known to regulate chaperone function were used in assembly assays using AP-MS. The results revealed that one of these drugs, riluzole, a compound previously approved by the FDA to treat some forms of ALS, positively affects Pol III assembly with the variant POLR3B R103H [6]. Because riluzole does not have a similar stimulatory effect when using other variants, Deep-SCP analysis appears to be even more necessary to generate additional information for selecting more potent compounds. For example, other benzothiazoles chemically similar to riluzole constitute strong candidates for the discovery of drugs targeting disease mechanisms in POLR3-HLD. Other compounds previously shown to positively affect molecular chaperones or co-chaperones, such as components of the Particle for Arrangement of Quaternary structure or PAQosome (see [6]), are also interesting candidates for therapy development.

Leukodystrophy-causing genes, such as the genes encoding faulty Pol III subunits, are putative targets for gene therapy. However, there are limitations in gene therapy approaches, including the size of the given gene, which affects its ability to be packaged within an adeno-associated vector, the challenge of efficiently targeting the most diseased cells, and in some cases, the difficulty of effectively crossing the blood–brain barrier or ensuring widespread distribution beyond the injection site. Deep-SCP will be used to identify proteins that likely play an early role in the disease. Their roles will be validated, leading to additional targets for therapy development using, for example, small molecules, which will be amenable to use in the clinic in a timely fashion and could potentially be used in combination with other therapeutic approaches, such as gene therapy, to ensure the targeting of all cell types and increase the therapeutic benefits. 

Detection by exome sequencing of disease-causing variants in patients (or their parents) is an important step for early detection. However, it is not always possible to link mutations specifically to the speed or severity of disease progression. We expect that our single-cell proteomics analysis will provide useful information not only to stratify cohorts but also to engage in meaningful drug discovery.

### 2.4. Collaborate

Leukodystrophies encompass a collection of individually rare diseases, with a collective incidence of approximately 1:4700 [21]. Due to their low prevalence, research is often limited by the paucity of disease-relevant patient tissue. To circumvent these issues, we have established a collaborative platform that encompasses multiple institutions with a focus on studying hypomyelinating leukodystrophies. Our platform was designed to seamlessly transition between the bedside and the bench, as research is directly informed by patient needs and data, with the ultimate goal of developing effective interventions and therapies. By leveraging animal and cell models specific to these leukodystrophies, we aim to further develop our proteomic pipeline to improve our understanding of disease mechanisms. We trust that Deep-SCP represents a powerful tool to characterize changes in physiological, clinical, and pharmacological conditions.

## 3. Conclusions and Future Prospects

Targeting the cells most susceptible to disease manifestations is ideal to study pathogenesis and develop novel therapies, increasing the likelihood of seamless transition to the clinic. This pioneering work employing cell-based interception and precision medicine in the study of leukodystrophies will serve as a foundational model for advancing therapeutics in other neurodegenerative diseases. 

## Data Availability

Not applicable.

## Abbreviations

Affinity purification coupled to mass spectrometry (AP-MS), Arginyl-tRNA synthetase (RARS), Asparagine (Asn), Aspartyl-tRNA synthetase (DARS), Deoxyribonucleic acid (DNA), Fluorescence-activated cell sorting (FACS), Food and Drug Administration (FDA), Glutamyl-prolyl-tRNA synthetase 1 (EPRS1), Hypomyelination, Hypodontia, and Hypogonadotropic Hypogonadism leukodystrophy (4H leukodystrophy), Induced pluripotent stem cell (iPSC), Oligodendrocyte precursor cells (OPCs), POLR3-related leukodystrophy (POLR3-HLD), Posttranslational modifications (PTMs), Principal component analysis (PCA), Ribonucleic acid (RNA), Ribosomal RNA (rRNA), RNA polymerase I subunit A (POLR1A), RNA polymerase III (POLR3 or Pol III), Single-cell (SC), Single-cell proteomics (SCP), Single-cell RNA sequencing (sc-RNAseq), Tandem Mass Tag (TMT), Threonine (Thr), Transfer RNA (tRNA), Valyl-tRNA synthetase 1 (VARS1).

## References

[1] LifeTime. Biomedical Research Initiative. https://lifetime-initiative.eu.

[2] Rajewsky N., Almouzni G., Gorski S.A., Aerts S., Amit I., Bertero M.G., Bock C., Bredenoord A.L., Cavalli G., Chiocca S. (2020). LifeTime and improving European healthcare through cell-based interceptive medicine. Nature.

[3] 37TrilllionCells. https://37TrillionCells.com.

[4] Thiffault I., Wolf N.I., Forget D., Guerrero K., Tran L.T., Choquet K., Lavallee-Adam M., Poitras C., Brais B., Yoon G. (2015). Recessive mutations in POLR1C cause a leukodystrophy by impairing biogenesis of RNA polymerase III. Nat. Commun..

[5] Choquet K., Forget D., Meloche E., Dicaire M.J., Bernard G., Vanderver A., Schiffmann R., Fabian M.R., Teichmann M., Coulombe B. (2019). Leukodystrophy-associated POLR3A mutations down-regulate the RNA polymerase III transcript and important regulatory RNA BC200. J. Biol. Chem..

[6] Pinard M., Dastpeyman S., Poitras C., Bernard G., Gauthier M.S., Coulombe B. (2022). Riluzole partially restores RNA polymerase III complex assembly in cells expressing the leukodystrophy-causative variant POLR3B R103H. Mol. Brain.

[7] Yeo G.H.T., Saksena S.D., Gifford D.K. (2021). Generative modeling of single-cell time series with PRESCIENT enables prediction of cell trajectories with interventions. Nat. Commun..

[8] Vanderver A., Prust M., Tonduti D., Mochel F., Hussey H.M., Helman G., Garbern J., Eichler F., Labauge P., Aubourg P. (2015). Case definition and classification of leukodystrophies and leukoencephalopathies. Mol. Genet. Metab..

[9] Schiffmann R., van der Knaap M.S. (2009). Invited article: An MRI-based approach to the diagnosis of white matter disorders. Neurology.

[10] Steenweg M.E., Vanderver A., Blaser S., Bizzi A., de Koning T.J., Mancini G.M., van Wieringen W.N., Barkhof F., Wolf N.I., van der Knaap M.S. (2010). Magnetic resonance imaging pattern recognition in hypomyelinating disorders. Brain.

[11] Coulombe B., Derksen A., La Piana R., Brais B., Gauthier M.S., Bernard G. (2021). POLR3-related leukodystrophy: How do mutations affecting RNA polymerase III subunits cause hypomyelination?. Fac. Rev..

[12] Adang L.A., Sherbini O., Ball L., Bloom M., Darbari A., Amartino H., DiVito D., Eichler F., Escolar M., Evans S.H. (2017). Revised consensus statement on the preventive and symptomatic care of patients with leukodystrophies. Mol. Genet. Metab..

[13] Keller S.R., Mallack E.J., Rubin J.P., Accardo J.A., Brault J.A., Corre C.S., Elizondo C., Garafola J., Jackson-Garcia A.C., Rhee J. (2021). Practical Approaches and Knowledge Gaps in the Care for Children With Leukodystrophies. J. Child. Neurol..

[14] Bernard G., Chouery E., Putorti M.L., Tetreault M., Takanohashi A., Carosso G., Clement I., Boespflug-Tanguy O., Rodriguez D., Delague V. (2011). Mutations of POLR3A encoding a catalytic subunit of RNA polymerase Pol III cause a recessive hypomyelinating leukodystrophy. Am. J. Hum. Genet..

[15] Tetreault M., Choquet K., Orcesi S., Tonduti D., Balottin U., Teichmann M., Fribourg S., Schiffmann R., Brais B., Vanderver A. (2011). Recessive mutations in POLR3B, encoding the second largest subunit of Pol III, cause a rare hypomyelinating leukodystrophy. Am. J. Hum. Genet..

[16] Macintosh J., Perrier S., Pinard M., Tran L.T., Guerrero K., Prasad C., Prasad A.N., Pastinen T., Thiffault I., Coulombe B. (2023). Biallelic pathogenic variants in POLR3D alter tRNA transcription and cause a hypomyelinating leukodystrophy: A case report. Front. Neurol..

[17] Mendes M.I., Gutierrez Salazar M., Guerrero K., Thiffault I., Salomons G.S., Gauquelin L., Tran L.T., Forget D., Gauthier M.S., Waisfisz Q. (2018). Bi-allelic Mutations in EPRS, Encoding the Glutamyl-Prolyl-Aminoacyl-tRNA Synthetase, Cause a Hypomyelinating Leukodystrophy. Am. J. Hum. Genet..

[18] Friedman J., Smith D.E., Issa M.Y., Stanley V., Wang R., Mendes M.I., Wright M.S., Wigby K., Hildreth A., Crawford J.R. (2019). Biallelic mutations in valyl-tRNA synthetase gene VARS are associated with a progressive neurodevelopmental epileptic encephalopathy. Nat. Commun..

[19] Lemire G., Ito Y.A., Marshall A.E., Chrestian N., Stanley V., Brady L., Tarnopolsky M., Curry C.J., Hartley T., Mears W. (2021). ABHD16A deficiency causes a complicated form of hereditary spastic paraplegia associated with intellectual disability and cerebral anomalies. Am. J. Hum. Genet..

[20] Spahr A., Rosli Z., Legault M., Tran L.T., Fournier S., Toutounchi H., Darbelli L., Madjar C., Lucia C., St-Jean M.L. (2021). The LORIS MyeliNeuroGene rare disease database for natural history studies and clinical trial readiness. Orphanet J. Rare Dis..

[21] Soderholm H.E., Chapin A.B., Bayrak-Toydemir P., Bonkowsky J.L. (2020). Elevated Leukodystrophy Incidence Predicted From Genomics Databases. Pediatr. Neurol..

[22] Dorboz I., Dumay-Odelot H., Boussaid K., Bouyacoub Y., Barreau P., Samaan S., Jmel H., Eymard-Pierre E., Cances C., Bar C. (2018). Mutation in POLR3K causes hypomyelinating leukodystrophy and abnormal ribosomal RNA regulation. Neurol. Genet..

[23] Wolf N.I., Vanderver A., van Spaendonk R.M., Schiffmann R., Brais B., Bugiani M., Sistermans E., Catsman-Berrevoets C., Kros J.M., Pinto P.S. (2014). Clinical spectrum of 4H leukodystrophy caused by POLR3A and POLR3B mutations. Neurology.

[24] Perrier S., Michell-Robinson M.A., Bernard G. (2020). POLR3-Related Leukodystrophy: Exploring Potential Therapeutic Approaches. Front. Cell Neurosci..

[25] Choquet K., Pinard M., Yang S., Moir R.D., Poitras C., Dicaire M.J., Sgarioto N., Lariviere R., Kleinman C.L., Willis I.M. (2019). The leukodystrophy mutation Polr3b R103H causes homozygote mouse embryonic lethality and impairs RNA polymerase III biogenesis. Mol. Brain.

[26] Ramsay E.P., Abascal-Palacios G., Daiss J.L., King H., Gouge J., Pilsl M., Beuron F., Morris E., Gunkel P., Engel C. (2020). Structure of human RNA polymerase III. Nat. Commun..

[27] Girbig M., Misiaszek A.D., Vorlander M.K., Lafita A., Grotsch H., Baudin F., Bateman A., Muller C.W. (2021). Cryo-EM structures of human RNA polymerase III in its unbound and transcribing states. Nat. Struct. Mol. Biol..

[28] Taft R.J., Vanderver A., Leventer R.J., Damiani S.A., Simons C., Grimmond S.M., Miller D., Schmidt J., Lockhart P.J., Pope K. (2013). Mutations in DARS cause hypomyelination with brain stem and spinal cord involvement and leg spasticity. Am. J. Hum. Genet..

[29] Wolf N.I., Salomons G.S., Rodenburg R.J., Pouwels P.J., Schieving J.H., Derks T.G., Fock J.M., Rump P., van Beek D.M., van der Knaap M.S. (2014). Mutations in RARS cause hypomyelination. Ann. Neurol..

[30] Misceo D., Lirussi L., Stromme P., Sumathipala D., Guerin A., Wolf N.I., Server A., Stensland M., Dalhus B., Tolun A. (2023). A homozygous POLR1A variant causes leukodystrophy and affects protein homeostasis. Brain.

[31] Kara B., Koroglu C., Peltonen K., Steinberg R.C., Maras Genc H., Holtta-Vuori M., Guven A., Kanerva K., Kotil T., Solakoglu S. (2017). Severe neurodegenerative disease in brothers with homozygous mutation in POLR1A. Eur. J. Hum. Genet..

[32] Perrier S., Gauquelin L., Fallet-Bianco C., Dishop M.K., Michell-Robinson M.A., Tran L.T., Guerrero K., Darbelli L., Srour M., Petrecca K. (2020). Expanding the phenotypic and molecular spectrum of RNA polymerase III-related leukodystrophy. Neurol. Genet..

[33] Perrier S., Gauquelin L., Wambach J.A., Bernard G. (2022). Distinguishing severe phenotypes associated with pathogenic variants in POLR3A. Am. J. Med. Genet. A.

[34] DeGasperis S.M., Bernard G., Wolf N.I., Miller E., Pohl D. (2020). 4H leukodystrophy: Mild clinical phenotype and comorbidity with multiple sclerosis. Neurol. Genet..

[35] Michell-Robinson M.A., Watt K.E.N., Grouza V., Macintosh J., Pinard M., Tuznik M., Chen X., Darbelli L., Wu C.L., Perrier S. (2023). Hypomyelination, hypodontia and craniofacial abnormalities in a Polr3b mouse model of leukodystrophy. Brain.

[36] Merheb E., Cui M.H., DuBois J.C., Branch C.A., Gulinello M., Shafit-Zagardo B., Moir R.D., Willis I.M. (2021). Defective myelination in an RNA polymerase III mutant leukodystrophic mouse. Proc. Natl. Acad. Sci. USA.

[37] Macintosh J., Michell-Robinson M., Chen X., Bernard G. (2023). Decreased RNA polymerase III subunit expression leads to defects in oligodendrocyte development. Front. Neurosci..

[38] Kuhn S., Gritti L., Crooks D., Dombrowski Y. (2019). Oligodendrocytes in Development, Myelin Generation and Beyond. Cells.

[39] Budnik B., Levy E., Harmange G., Slavov N. (2018). SCoPE-MS: Mass spectrometry of single mammalian cells quantifies proteome heterogeneity during cell differentiation. Genome Biol..

[40] Petelski A.A., Emmott E., Leduc A., Huffman R.G., Specht H., Perlman D.H., Slavov N. (2021). Multiplexed single-cell proteomics using SCoPE2. Nat. Protoc..

[41] Specht H., Emmott E., Petelski A.A., Huffman R.G., Perlman D.H., Serra M., Kharchenko P., Koller A., Slavov N. (2021). Single-cell proteomic and transcriptomic analysis of macrophage heterogeneity using SCoPE2. Genome Biol..

[42] Tyanova S., Temu T., Cox J. (2016). The MaxQuant computational platform for mass spectrometry-based shotgun proteomics. Nat. Protoc..

[43] Huffman R.G., Chen A., Specht H., Slavov N. (2019). DO-MS: Data-Driven Optimization of Mass Spectrometry Methods. J. Proteome Res..

[44] UniProt C. (2021). UniProt: The universal protein knowledgebase in 2021. Nucleic Acids Res.

[45] Lever J., Krzywinski M., Altman N. (2017). Principal component analysis. Nat. Methods.

[46] Sangster M., Shahriar S., Niziolek Z., Carisi M.C., Lewandowski M., Budnik B., Grishchuk Y. (2023). Brain cell type specific proteomics approach to discover pathological mechanisms in the childhood CNS disorder mucolipidosis type IV. Front. Mol. Neurosci..

[47] Piscopo V.E.C., Chapleau A., Blaszczyk G.J., Sirois J., You Z., Soubannier V., Chen C.X., Bernard G., Antel J.P., Durcan T.M. (2024). The use of a SOX10 reporter toward ameliorating oligodendrocyte lineage differentiation from human induced pluripotent stem cells. Glia.

[48] Douvaras P., Fossati V. (2015). Generation and isolation of oligodendrocyte progenitor cells from human pluripotent stem cells. Nat. Protoc..

[49] Thomas R.A., Sirois J., Li S., Gestin A., Piscopo V.E., Lépine P., Mathur M., Chen C.X., Soubannier V., Goldsmith T.M. (2023). Fon. CelltypeR: A flow cytometry pipeline to annotate, characterize and isolate single cells from brain organoids. BioRxiv.

[50] Chamling X., Kallman A., Fang W., Berlinicke C.A., Mertz J.L., Devkota P., Pantoja I.E.M., Smith M.D., Ji Z., Chang C. (2021). Single-cell transcriptomic reveals molecular diversity and developmental heterogeneity of human stem cell-derived oligodendrocyte lineage cells. Nat. Commun..

[51] Frazel P.W., Labib D., Fisher T., Brosh R., Pirjanian N., Marchildon A., Boeke J.D., Fossati V., Liddelow S.A. (2023). Longitudinal scRNA-seq analysis in mouse and human informs optimization of rapid mouse astrocyte differentiation protocols. Nat. Neurosci..

[52] Zheng C., Tu C., Wang J., Yu Y., Guo X., Sun J., Sun J., Cai W., Yang Q., Sun T. (2023). Deciphering Oligodendrocyte Lineages in the Human Fetal Central Nervous System Using Single-Cell RNA Sequencing. Mol. Neurobiol..

[53] Dennis D.J., Wang B.S., Karamboulas K., Kaplan D.R., Miller F.D. (2024). Single-cell approaches define two groups of mammalian oligodendrocyte precursor cells and their evolution over developmental time. Stem Cell Rep..

[54] Jakel S., Agirre E., Mendanha Falcao A., van Bruggen D., Lee K.W., Knuesel I., Malhotra D., Ffrench-Constant C., Williams A., Castelo-Branco G. (2019). Altered human oligodendrocyte heterogeneity in multiple sclerosis. Nature.

[55] Marques S., Zeisel A., Codeluppi S., van Bruggen D., Mendanha Falcao A., Xiao L., Li H., Haring M., Hochgerner H., Romanov R.A. (2016). Oligodendrocyte heterogeneity in the mouse juvenile and adult central nervous system. Science.

[56] Valihrach L., Matusova Z., Zucha D., Klassen R., Benesova S., Abaffy P., Kubista M., Anderova M. (2022). Recent advances in deciphering oligodendrocyte heterogeneity with single-cell transcriptomics. Front. Cell Neurosci..

[57] Yao Z., van Velthoven C.T.J., Kunst M., Zhang M., McMillen D., Lee C., Jung W., Goldy J., Abdelhak A., Aitken M. (2023). A high-resolution transcriptomic and spatial atlas of cell types in the whole mouse brain. Nature.

[58] Ramazi S., Zahiri J. (2021). Posttranslational modifications in proteins: Resources, tools and prediction methods. Database.

[59] Woo J., Clair G.C., Williams S.M., Feng S., Tsai C.F., Moore R.J., Chrisler W.B., Smith R.D., Kelly R.T., Pasa-Tolic L. (2022). Three-dimensional feature matching improves coverage for single-cell proteomics based on ion mobility filtering. Cell Syst..

[60] Rosenberger F.A., Thielert M., Strauss M.T., Schweizer L., Ammar C., Madler S.C., Metousis A., Skowronek P., Wahle M., Madden K. (2023). Spatial single-cell mass spectrometry defines zonation of the hepatocyte proteome. Nat. Methods.

[61] Straubhaar J., D’Souza A., Niziolek Z., Budnik B. (2024). Single cell proteomics analysis of drug response shows its potential as a drug discovery platform. Mol. Omics.

[62] de la Fuente A.G., Queiroz R.M.L., Ghosh T., McMurran C.E., Cubillos J.F., Bergles D.E., Fitzgerald D.C., Jones C.A., Lilley K.S., Glover C.P. (2020). Changes in the Oligodendrocyte Progenitor Cell Proteome with Ageing. Mol. Cell Proteomics.

[63] Gargareta V.I., Reuschenbach J., Siems S.B., Sun T., Piepkorn L., Mangana C., Spate E., Goebbels S., Huitinga I., Mobius W. (2022). Conservation and divergence of myelin proteome and oligodendrocyte transcriptome profiles between humans and mice. Elife.

[64] Feng L., Chao J., Zhang M., Pacquing E., Hu W., Shi Y. (2023). Developing a human iPSC-derived three-dimensional myelin spheroid platform for modeling myelin diseases. iScience.

[65] Hartl F.U., Bracher A., Hayer-Hartl M. (2011). Molecular chaperones in protein folding and proteostasis. Nature.

[66] Calamini B., Morimoto R.I. (2012). Protein homeostasis as a therapeutic target for diseases of protein conformation. Curr. Top. Med. Chem..

